# Psychological problems and work ability in unemployed people diagnosed with a mental illness

**DOI:** 10.1177/00207640241300959

**Published:** 2024-11-28

**Authors:** Felix S Hussenoeder, Maria Koschig, Alexander Pabst, Katharina Gatzsche, Luise Bieler, Mathias Alberti, Steffi G Riedel-Heller, Katarina Stengler, Ines Conrad

**Affiliations:** 1 Institute of Social Medicine, Occupational Health and Public Health, Leipzig University, Germany; 2 Helios Park-Klinikum - Clinic for Psychiatry, Psychosomatics and Psychotherapy, Leipzig, Germany

**Keywords:** Work ability, mental health, unemployment, psychological problems

## Abstract

**Background:**

Research shows that unemployed individuals are more often affected by mental illness, and that mental illness can impede an individual’s work ability, creating a significant obstacle to (re)entering the workforce.

**Aims:**

The objective of this study is to examine the relationships between psychological symptoms and work ability in unemployed individuals with mental illnesses (UMIs). This will enable us to identify the symptoms that are most relevant for future research and application.

**Method:**

Our study is based on a sample from the project LIPSY, which aims to maintain and/or restore the employability of UMIs. We conducted regression analyses with the outcome variable work ability in a sample of unemployed individuals with a mental illness (ICD-10: F-diagnosis). In the initial analysis, age, gender, education, and cohabitation status were used as predictors; in the final model, all nine symptom dimensions (SCL-90) were added, that is, (1) Somatization, (2) Obsessive-Compulsive, (3) Interpersonal Sensitivity, (4) Depression, (5) Anxiety, (6) Anger-Hostility, (7) Phobic Anxiety, (8) Paranoid Ideation, and (9) Psychoticism.

**Results:**

Our sample comprised 402 participants, with a mean age of 35.7 years, 52.5% were female. In the final analysis, we found significant positive associations between education, Paranoid Ideation, and work ability, and a significant negative one between Depression and work ability, but no other significant associations.

**Conclusions:**

The elevated scores on all SCL-90 dimensions, along with the associations between two dimensions and work ability, underscore the need for psychological screening, diagnosis, prevention, and therapy (Depression) as well as additional public health attention and research (Paranoid Ideation) in this high-risk population.

## Introduction

Research shows that unemployed individuals are more often affected by mental illness, and that unemployment and mental illness can reinforce each other (Dehn et al., 2023; Hakulinen et al., 2019; Virgolino et al., 2022). In addition, mental illness can have a negative effect on the ability to work (Cadiz et al., 2019; Karpov et al., 2017; Weber et al., 2021), creating a significant obstacle for unemployed individuals with mental illnesses (UMIs) to (re)enter the workforce. Consequently, their ability to reap the mental health benefits associated with workplace (re)integration is hindered (Heinz et al., 2018; Modini et al., 2016; van der Noordt et al., 2014). Furthermore, research indicates that a loss of work ability not only increases the risk of depression, but that this effect is especially pronounced in individuals who are unemployed (Lee et al., 2017).

Because mental illnesses are complex syndromes that vary from person to person (Cavanagh et al., 2017; Fried et al., 2017; Gordon & Heimberg, 2011), it is often difficult to identify which symptoms are responsible for the relationship between illness and work ability. In order to gain a better understanding of these relationships, it is therefore important to look at the symptom rather than the syndrome level. Hence, the aim of our study is to examine the relationships between psychological problems and symptoms of psychopathology, that is, the nine symptom dimensions (1) somatization, (2) obsessive-compulsive, (3) interpersonal sensitivity, (4) depression, (5) anxiety, (6) anger-hostility, (7) phobic anxiety, (8) paranoid ideation, and (9) psychoticism (Franke, 2014), and work ability.

Research findings indicate that the average SCL-90 symptom score is negatively correlated with work ability in Chinese coal workers (Yi et al., 2022) and positively correlated with job stress, but not with job satisfaction, in Korean marine officers (J. H. Kim & Jang, 2016). Our study adds to the literature by analyzing relationships on the symptom level in a highly under-researched group, that is, UMIs. The objective is to identify the symptoms that are most pertinent to future research and to potential applications.

## Methods

Our study utilizes a sample derived from LIPSY (‘Leipzig – Individual Placement and Support for people with mental illnesses’), a project dedicated to maintaining and/ or restoring the employability of UMIs who receive means-tested benefits. The project’s goal is to facilitate participants’ (re)integration into the labor market through targeted advice and, in certain cases, personalized coaching (Hussenoeder et al., 2021). The evaluation process is conducted using self-assessment questionnaires administered at four different points in time over the course of 18 months, beginning with a comprehensive psychiatric assessment at baseline. For this study, we analyzed the initial 600 baseline questionnaires.

Participants included in our analysis were diagnosed with at least one mental illness by a professional psychologist (ICD-10 code: F). After data cleaning and the exclusion of questionnaires with missing values, the final analysis sample comprised 402 individuals.

## Ethics

The study is in accordance with the Helsinki Declaration of 1975 (revised in 2008) and received approval from the Ethics Committee of the University of Leipzig. Written informed consent was obtained from all participants.

## Measures

### Socio-Demographic Variables

Participants were asked to provide data on a number of demographic characteristics, including age, gender, level of education, and whether or not they were cohabiting with a partner. Education was classified into the following four categories: (1) no school leaving certificate, (2) low (secondary school leaving certificate), (3) medium (intermediate or 10th-grade polytechnic secondary school leaving certificate), and (4) high (advanced technical college entrance qualification, technical secondary school leaving certificate, general or specialized higher education entrance qualification/Abitur).

### Symptom Checklist

The SCL-90-S is a self-report measure that is used to assess the subjectively perceived impairment due to physical and psychological symptoms that have occurred over the past 7 days (Franke, 2014). The 90 items belong to the following nine dimensions: (1) Somatization, (2) Obsessive-Compulsive, (3) Interpersonal Sensitivity, (4) Depression, (5) Anxiety, (6) Anger-Hostility, (7) Phobic Anxiety, (8) Paranoid Ideation, and (9) Psychoticism. The scales range from 0 to 4. The Global Severity Index (GSI), which is calculated as the average score of all 90 items, reflects the subject’s overall level of distress. The SCL-90-S is a slightly revised version of the SCL-90-R, and the results of both versions are comparable (Hergert et al., 2014).

### Work Ability

We used the work ability item from the Work Ability Index (WAI; INQA-WAI-Netzwerk, 2017), where individuals can indicate their ability to work on a scale from 0 to 10, with higher scores denoting elevated levels of work ability. The item has been shown to be a suitable measure for the assessment of work ability (Ebener & Hasselhorn, 2019).

## Statistical Analyses

We used IBM SPSS (version 27) for the statistical analyses. The initial regression analysis on work ability included the predictors age, gender, education, and cohabitation status as independent variables. In the final regression we added (1) Somatization, (2) Obsessive-Compulsive, (3) Interpersonal Sensitivity, (4) Depression, (5) Anxiety, (6) Anger-Hostility, (7) Phobic Anxiety, (8) Paranoid Ideation, and (9) Psychoticism as predictors. The analyses were performed with robust standard errors (Hayes & Cai, 2007).

## Results

Our sample comprised 402 UMIs, with a mean age of 35.7 years, the sample was 52.5% female. Table 1 provides an overview of the general characteristics of the sample.

**Table 1.** General characteristics of the study population.

Total group (*N* = 402)	
Age (years)	35.7 (11.1)
Gender (‘female’)	211 (52.5%)
*Education*	
No school leaving certificate	44 (10.9%)
Low (secondary general school)	101 (25.1%)
Medium (intermediate/ polytechnic secondary school)	160 (39.8%)
High (technical secondary school, university entrance qualification)	97 (24.1%)
Living with a partner (‘yes’)	69 (17.2%)
*Symptom dimensions* (SCL-90; Range: 0–4)	
Somatization	1.1 (0.8)
Obsessive-Compulsive	1.6 (0.8)
Interpersonal Sensitivity	1.6 (0.9)
Depression	1.8 (0.8)
Anxiety	1.3 (0.8)
Anger-Hostility	1.1 (0.9)
Phobic Anxiety	1.3 (1.0)
Paranoid Ideation	1.3 (0.9)
Psychoticism	0.9 (0.6)
Global Severity Index^1^ (SCL-90; range: 0–4)	1.4 (0.6)
Work ability (WAI; range: 0–10)	3.3 (2.4)

*Note.* Continuous variables are given as mean (standard deviation); categorical variables are displayed as numbers (percentages).

^1^The Global Severity Index reflects the overall level of psychological distress a person is experiencing. It represents the average score over all 90 items from the SCL-90.

Table 2 presents both regression analyses, the first one with only control variables and the second one with control variables and predictors. In the first regression, there are significant negative associations between age, cohabitation status, and work ability, and a significant positive one between a high level of education and work ability. In the second regression, there are significant positive associations between a high level of education, Paranoid Ideation, and work ability, and a significant negative one between Depression and work ability. None of the other SCL-90 dimensions exhibited significant connections with the outcome.

**Table 2.** Prediction of work ability by control variables alone and by control variables and the nine SCL-90 dimensions (standardized regression coefficients; *N* = 402).

Variable	Work ability	
	β	*B*
Age	−.12**	−.10
Gender (‘female’)^1^	.08	−.02
*Education* (‘no certification’)		
Low	.03	.05
Moderate	.07	.10
High	.23**	.23**
Living with partner (‘no’)	−.10*	−.08
Somatization		−.13
Obsessive-Compulsive		.00
Interpersonal Sensitivity		−.07
Depression		−.25**
Anxiety		−.09
Anger-Hostility		.02
Phobic Anxiety		−.08
Paranoid Ideation		.16*
Psychoticism		.11
*R* ^2^	.07	.18

*Note*. Analyses were performed with robust standard errors. Values in bold letters show significant correlations.

^1^The category coded as ‘0’ (=reference category) is presented in parentheses.

* *p* ⩽ .05. **p ⩽ .01. ***p ⩽ .001.

## Discussion

Our results demonstrated significant positive connections between educational attainment, Paranoid Ideation, and work ability, and a significant inverse relationship between Depression and work ability; however, no significant associations were found between the remaining SCL-90 dimensions and work ability

The level of work ability in our sample (3.3) was found to be significantly lower than that observed in a representative sample of employees in Germany between the ages of 31 and 60 (work ability = 8.0; Freyer et al., 2019), and also markedly inferior to the value observed in a large sample of nurses in European countries (8.1; Ebener & Hasselhorn, 2019). The participants in our sample also exhibited lower values than those observed in unemployed or laid-off individuals from a Finnish study (7.4; Hult et al., 2018) and depressed individuals in a Swedish intervention study (5.8; Hange et al., 2017). A value of 2.0 was identified in a sample of patients with chronic pain (Zetterberg et al., 2023); however, it should be noted that the sample was recruited from secondary and tertiary care settings, and approximately half of the participants were either on long-term sick leave or pensioners (due to disability or early retirement). The results of these studies indicate that individuals in our sample experience a dual burden, encompassing both unemployment and mental illness, which collectively influence their work ability. This interpretation is corroborated by another study that demonstrates elevated levels of loneliness in a comparable sample (Hussenoeder et al., 2024). Furthermore, the literature suggests that work ability is a pivotal aspect when it comes to UMIs, particularly when considering that enhanced work ability is linked to an increased likelihood of returning to work (Kuijer et al., 2012; Ottiger et al., 2024) and to better mental health, quality of life, and later-life health (Nordstoga et al., 2019; Seitsamo et al., 2011; van Hoffen et al., 2021).

The level of Depression in our sample (1.8) was considerably higher than that observed in the general population (0.4; Schmitz et al., 2000). Moreover, our findings revealed a clear association between elevated levels of depression and diminished work ability, a result that aligns with previous studies on civil workers, breast cancer survivors, and patients with depression (Abdelrehim et al., 2023; Hange et al., 2017; S.-Y. Kim et al., 2022). Our findings are also consistent with prior studies that have demonstrated concurrent improvements in depression and work ability (Petersson et al., 2018) and have suggested causal interpretations in both directions (Cadiz et al., 2019; Lee et al., 2017; Weber et al., 2021).

Our results highlight the necessity for depression screening, diagnosis, and treatment in the UMI population, particularly given the established links between depression and a range of adverse health outcomes, from stress to suicide attempts and dementia (Dong et al., 2019; Fernández Fernández et al., 2023; Hussenoeder et al., 2022). Furthermore, it is recommended to implement active prevention strategies to overcome motivational barriers within the population (Staiger et al., 2017). Digital applications and internet-based measures may prove to be effective tools for providing accessible support at subclinical levels of depression (Hussenoeder, 2022; Wolff et al., 2023).

Paranoid Ideation refers to persistent thoughts of suspicion, mistrust, and the unfounded belief that one is being persecuted or targeted by others. The participants in our sample exhibited a score of 1.3, which is clearly above the German population level (0.4), and also above the value observed in a sample of German psychosomatic outpatients (Schmitz et al., 2000). This is problematic since Paranoid Ideation is associated with a range of mental health issues, including depression, anxiety, suicide attempts, social detachment, violence, and other forms of psychopathology (Coid et al., 2016; Na et al., 2019; Saarinen et al., 2022), which have broader implications for both individual and societal well-being. Furthermore, stress, worry, social detachment, and sleep disturbances, which are more prevalent among unemployed individuals and those with mental illnesses, can exacerbate Paranoid Ideation (Saarinen et al., 2022). In light of the literature, the observed positive association between Paranoid Ideation and work ability in our sample is somewhat unexpected. It is only possible to speculate about causal interpretations. For example, an increased Paranoid Ideation may contribute to a coping style that attributes problems associated with work ability to others rather than to oneself, thus resulting in a higher rating of work ability. Conversely, a high level of perceived work ability in conjunction with unemployment may prompt individuals to search for the root of their current situation outside of themselves, thereby leading to an increased Paranoid Ideation. Further research is required to examine these relationships in greater detail and either confirm or refute the proposed mechanisms.

In general, while paranoid ideation poses public health problems, it is nevertheless under-researched in comparison to other symptoms. A quick search with Google Scholar for ‘paranoid ideation’ on August 12, 2024 yielded only 43,200 results in comparison to 4,400,000 for ‘anxiety’ and 4,320,000 for ‘depression’. However, this figure is also lower than that for less established and narrower concepts, such as ‘interpersonal sensitivity’ (56,000) and ‘anger-hostility’ (45,800). Future research may facilitate the identification of risk groups for Paranoid Ideation and the development of measures and interventions.

Our results demonstrated no statistically significant associations between obsessive-compulsive, interpersonal sensitivity, anxiety, anger-hostility, phobic anxiety, psychoticism, and work ability. Nevertheless, our descriptive findings (Table 1) indicate that UMIs exhibit a higher burden on all nine SCL-90 dimensions when compared to the general population and to a sample of primary care patients (Schmitz et al., 2000). These elevated levels of SCL-90 domain scores may constitute mental health problems in and of themselves and may impact the reintegration of UMIs into the labor market. That is to say, even if they do not exhibit significant effects with regard to work ability, they should nevertheless be regarded as problematic and as potential goals for public health interventions.

This study has several strengths, including the utilization of a sample that has been significantly under-researched, with high public health relevance. However, the study also has some limitations that could be addressed by future research. For example, the cross-sectional nature of the dataset limits the ability to draw causal inferences, and the lack of individuals who are unemployed and healthy or employed and suffering from a mental illness represents an obstacle to attaining a more nuanced understanding of potential (over)additive effects.
